# SmartWoodID—an image collection of large end-grain surfaces to support wood identification systems

**DOI:** 10.1093/database/baad034

**Published:** 2023-05-13

**Authors:** Ruben De Blaere, Kévin Lievens, Dieter Van Hassel, Victor Deklerck, Tom De Mil, Wannes Hubau, Joris Van Acker, Nils Bourland, Jan Verwaeren, Jan Van den Bulcke, Hans Beeckman

**Affiliations:** Service of Wood Biology, Royal Museum for Central Africa, Leuvensesteenweg 13, Tervuren 3080, Belgium; Service of Wood Biology, Royal Museum for Central Africa, Leuvensesteenweg 13, Tervuren 3080, Belgium; Service of Wood Biology, Royal Museum for Central Africa, Leuvensesteenweg 13, Tervuren 3080, Belgium; Jodrell laboratory, Royal Botanic Gardens, Kew, Richmond, London TW9 3A, UK; TERRA Teaching and Research Center, Gembloux Agro-Bio Tech (Université de Liège), Passage des Déportés 2, Gembloux 5030, Belgium; Service of Wood Biology, Royal Museum for Central Africa, Leuvensesteenweg 13, Tervuren 3080, Belgium; UGent-Woodlab, Laboratory of Wood Technology, Department of Environment, Ghent University, Coupure Links 653, Gent 9000, Belgium; UGent-Woodlab, Laboratory of Wood Technology, Department of Environment, Ghent University, Coupure Links 653, Gent 9000, Belgium; Service of Wood Biology, Royal Museum for Central Africa, Leuvensesteenweg 13, Tervuren 3080, Belgium; UGent-KERMIT, Research Unit Knowledge-based, Predictive and Spatio-temporal Modelling, Department of Data Analysis and Mathematical Modelling, Ghent University, Coupure Links 653, Gent 9000, Belgium; UGent-Woodlab, Laboratory of Wood Technology, Department of Environment, Ghent University, Coupure Links 653, Gent 9000, Belgium; Service of Wood Biology, Royal Museum for Central Africa, Leuvensesteenweg 13, Tervuren 3080, Belgium

## Abstract

Wood identification is a key step in the enforcement of laws and regulations aimed at combatting illegal timber trade. Robust wood identification tools, capable of distinguishing a large number of timbers, depend on a solid database of reference material. Reference material for wood identification is typically curated in botanical collections dedicated to wood consisting of samples of secondary xylem of lignified plants. Specimens from the Tervuren Wood Collection, one of the large institutional wood collections around the world, are used as a source of tree species data with potential application as timber. Here, we present SmartWoodID, a database of high-resolution optical scans of the end-grain surfaces enriched with expert wood anatomical descriptions of macroscopic features. These can serve as annotated training data to develop interactive identification keys and artificial intelligence for computer vision–based wood identification. The first edition of the database consists of images of 1190 taxa, with a focus on potential timber species from the Democratic Republic of the Congo with at least four different specimens per species included.

**Database URL** https://hdl.handle.net/20.500.12624/SmartWoodID_first_edition

## Introduction

Illegal logging significantly impacts forests, posing a high risk of irreversible damage, particularly when exploiting populations of protected species. Thirty to ninety per cent of traded tropical timber is estimated to have been harvested illegally (1–3). Timber regulations (such as Forest Law Enforcement, Governance and Trade; European Union Timber Regulation; US Lacey Act and the Illegal Logging Prohibition Act in Australia) and the Convention on International Trade in Endangered Species (CITES) face implementation and enforcement challenges, as trade cannot be regulated without accurate assessment of origin and identity claims (4, 5). Therefore, fast and accurate wood identification systems are needed to properly implement timber regulations by verifying whether the traded species matches the species name on accompanying documents.

The most commonly used and affordable method for wood identification is the wood anatomical assessment. It involves observing tissues and cells at different scales and planes to identify the diagnostic features of a botanical taxon. Standardized features for identification have been published by the International Association of Wood Anatomists (IAWA) (6–11). The importance of standardized features is to ensure consistency in identification. Microscopic features, observed in a laboratory with specialized equipment, are typically used, but some macroscopic features observable with a hand lens can also be used in the field for faster identification (11, 12).

In addition to wood anatomical assessment, alternative methods for wood identification include Near-infrared spectroscopy (NIRS), genetic techniques and mass spectrometry such as Direct Analysis in Real-Time (DART) time-of-flight mass spectrometry (TOFMS). NIRS can potentially distinguish between species by differences in near-infrared absorption (13–15), although it still requires more development before becoming a common method in forensic research (16). Genetic techniques use deoxyribonucleic acid (DNA)-based approaches and can track individual logs throughout the supply chain (17), but extracting high-quality DNA from timber is difficult, especially when dried or processed (18–21). Mass spectrometry, specifically DART TOFMS, produces a chemical fingerprint indicative of a wood species, but challenges include the need for reference databases (15, 16, 22). Currently, efforts are being made, for example, by World Forest ID (5), to create large reference datasets on stable isotope ratios, DART TOFMS and other identification or harvest origin determination techniques, but they are still under construction.

To aid in wood species recognition, identification keys, including digital formats, can be useful due to the vast number of tree species, especially when the geographic origin is unknown (23–26). These keys allow for the observation of anatomical features and provide a list of matching species. Classification keys are advantageous for their speed and flexibility, with some allowing for a specified number of feature mismatches or required presence/absence of certain features (26, 27). Keys can be accessed online with large reference material or offline, suitable for remote locations such as local lumber mills in the tropics.

Computer vision (CV)-based wood identification is a method to automate the process of wood anatomical assessment and a possible solution to problems that untrained experts encounter. CV is a field of artificial intelligence (AI) that trains computers to interpret and understand the visual world (28–30). It relies on machine learning algorithms that use vast numbers of human-annotated reference images to distinguish timbers based on imagery. After successful training, the CV-based method can extract and use relevant features for timber identification. CV-based wood identification tools have potential for field deployment, enabling non-expert field workers to perform timber tracking. CV-based wood identification tools are suitable for field workers with less expertise in wood anatomy and have demonstrated their potential for real-world field deployment, for example, in Ghana (31).

**Figure 1.**
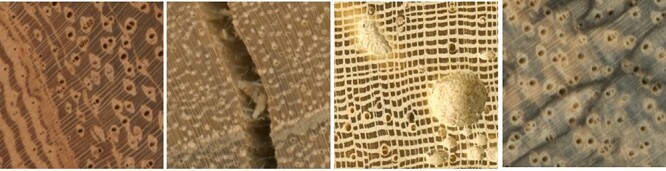
Examples of the intra-variability and anomalies that can be encountered on images of wood (end-grain surface). From left to right: (a) An example of the variability of wood anatomical features (such as axial parenchyma) on a single specimen. (b) An example of a possible anomaly on wood, a crack. (c) An example of a possible anomaly on wood, insect holes. (d) An example of a possible anomaly on wood, fungi damage. RubenDeBlaere©RMCA.

CV-based wood identification has the advantage of fast and easy application but faces risks due to the highly variable nature of wood, which exhibits inter- and intra-specific variability and anomalies like cracks, insect holes and fungal damage that can hinder the recognition of wood features (Figure 1). This makes it challenging to train machine learning models for field-based wood identification.

To build an accurate identification tool, a database with typical species features and sufficient details is needed, including visual and/or textual information on multiple specimens to consider biological variations. Online databases, such as macroHOLZdata (32) and the Atlas of Macroscopic Wood Identification (33), provide macroscopic anatomical descriptions of wood, but they may not cover all intra-specific variability of anatomical features that can occur in wood. The most complete online database for timber identification is InsideWood, a wood anatomy reference, research and teaching tool, containing wood anatomical descriptions of wood based on the IAWA Lists of Microscopic Features for Hardwood and Softwood Identification accompanied by a collection of photomicrographs (6, 9, 34). This database has a global scope and therefore incorporates timbers from all over the world, having >9400 wood anatomical descriptions of fossil and modern woody dicots, representing >10 000 species and 200 plant families, accompanied by >50 000 images of both microscopic and macroscopic features (34). Still, while this database serves a key purpose as a reference resource, this does not mean that its descriptions cover all intra-species variability of anatomical features that can occur in wood. Wood is variable and requires descriptions of large areas on multiple specimens. Additionally, although this database is relatively large, certainly not all woody species are represented with a sufficient number of individuals. It is the paucity of large databases that cover the variability of wood anatomical features, which is the main obstacle currently faced when building classification keys or machine learning models (29, 35). This paucity of large-quality datasets stems from the difficulty of acquiring sufficient wood specimens that give a faithful representation of all species and their variability in a geographically delineated area.

We built the first edition of SmartWoodID, an image database of end-grain wood that includes macroscopic features and anomalies, such as cracks, fungi damage and insect damage, and their variability along a radial gradient (i.e. a gradient from pith to bark). This first edition focuses on tree species from the Democratic Republic of the Congo (DRC) and serves as annotated training data for developing classification keys and AI for CV-based wood identification. SmartWoodID will be gradually extended with images of timbers from other continents in the coming years. The resulting database can also provide unique insights into the occurrence of characteristics, for example, within families.

## Material and methods

### The choice of the end-grain surface

A full wood anatomical assessment is performed through observations on three principal sections at different magnifications, more specifically the cross-section, radial section and tangential section. The end-grain surface or cross-section is perpendicular to the axial direction of growth or the grain of a piece of wood. This makes that it is relatively easy to find for non-specialists. Additionally, it is often easier to find a clear example of this section in comparison to the radial and tangential sections as those depend on the orientation of the rays, which might not be optimal for the anatomical assessment on a piece of sawn timber. Finally, many anatomical features (with value for wood identification) are visible on the end-grain surface. For this reason, we chose to digitize the cross-sections of wood specimens.

### Delineating a set area for the first edition

Given the complex and variable structure of a wooden tissue and the large number of tree species worldwide, digitizing a large amount of wood specimens is a long-term process. The SmartWoodID database will therefore gradually be extended over the following years with images and annotated materials from wood species from all over the world. This will be done in several editions containing images and annotated materials from large geographically delineated regions, in order to ensure that the data are available and usable for research on entire biomes rather than adding species *ad hoc*.

The first edition of the database focuses on the tree species of Central Africa and more specifically the tree species from the DRC. The following definition of trees is used here: perennial woody seed plants with a single dominant stem that is self-supporting and undergoes secondary growth. The DRC was selected because the vast area of the country and the different forest biomes make the DRC rich in tree species and thereby representative of species richness for all countries in the Congo Basin and ensure that many timbers or potentially commercial tree taxa of tropical Africa are included.

**Figure 2.**
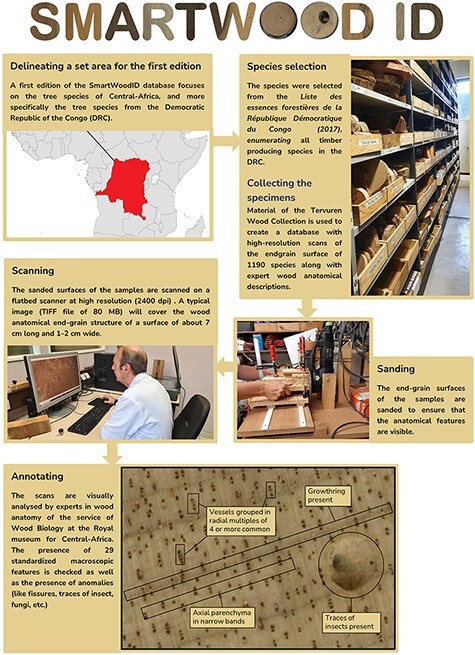
A flowchart showing the procedure of building the database.

An overview of the procedure, explaining the material and methods, is shown as a flowchart in Figure 2.

### Species selection

A list with accepted species names has been created according to the World Checklist of Vascular Plants (WCVP) (36) and the African Plant Database (APD) (37), providing information on accepted name status and synonymy. In this research, we will regard not only species but also accepted varieties and subspecies. All instances of species, varieties and subspecies shall be named ‘species and lower taxa’ from this point onwards to improve the ease of reading.

Two lists were used as a reference for all current and potential timbers in the DRC. The first is the list of the DRC forest administration *Direction Inventaire et Aménagement Forestiers* (DIAF), summarizing all tree species and lower taxa present in DRC forests, along with an indication of their current economic value (38). The second list is extracted from the RAINBIO database, from which all tree species and lower taxa were selected that occur in the DRC (39). The accepted name status of species and lower taxa names was cross-referenced and harmonized with the WCVP (36) as a reference, using a custom-developed Python script. The number of species and lower taxa for which no direct match to WCVP was found was checked against the APD (37), a curated list of >205 456 names of African plants with their nomenclatural status being a product of a collaboration between the South African National Biodiversity Institute, the *Conservatoire et Jardin botaniques de la Ville de Genève*, Tela Botanica and the Missouri Botanical Garden. Taxa that did not match WCVP or APD were reviewed manually, and any misspellings or synonyms that had not been automatically detected were corrected. Having standardized taxonomic names, any records from species and lower taxa that did not meet our working definition of trees—perennial woody seed plants with a single dominant stem that is self-supporting and undergoes secondary growth—were manually removed from the database. This included removing all ferns, palms, lianas, strangler figs, bamboos, pandans, as well as a number of shrub species and lower taxa that rarely exceed 2 m in height and are generally multi-stemmed. Finally, the accepted name of each species and lower taxon was used to check their presence in the Tervuren Wood Collection. The list contains also introduced species and lower taxa.

The International Union for Conservation of nature (IUCN) Red List of Threatened Species (46) was used to add information on Red List Categories and population trends. This was done to give an overview of the threatened tree species and lower taxa in the DRC and give a perspective on the threatened nature of commercial timbers, which are provided by the indication of the economic value of those species and lower taxa.

Information on the occurrence of those species and lower taxa in different vegetation types was also added by combining the geographical occurrence data in the RAINBIO database (39) with the geographical distribution of vegetation types in the Global Land Cover Map 2000 (GLC 2000 map) (40). Twenty-seven different classes are used in the GLC 2000 map to classify African vegetation. These classes were combined into larger classes based on research by Fritz (41) and consist of closed forests, edaphic forests, altitudinal forests, woodlands, shrub lands, savannahs, deserts, water bodies and urban areas. An overview of all classes is given in Table 1.

**Table 1.** Overview of all vegetation classes according to GLC 2000

Merged classes	Vegetation class (GLC 2000)
Altitudinal forest	Submontane forest (900–1500 m) Montane forest (>1500 m)
Background^a^	Background
Closed forest	Closed evergreen lowland forest Degraded evergreen lowland forest Mosaic forest/croplands Mosaic forest/savannah
Desert	Sandy desert and dunes Stony desert Bare rock Salt hardpans
Edaphic forest	Swamp forest Mangrove Swamp bush land and grassland
Savannah	Closed grassland Open grassland with sparse shrubs Open grassland Sparse grassland Croplands (>50%)
Shrub land	Deciduous shrub land with sparse trees Open deciduous shrub land Croplands with open woody vegetation
Urban areas	Irrigated croplands Tree crops Cities
Water bodies	Water bodies
Woodland	Closed deciduous forest Deciduous woodland

a This class does not occur in the DRC and shall therefore not be covered in the results or discussion sections.

The GLC 2000 map of Africa (product 2, version 5.0) was imported in QGIS 3.24.3 along with the occurrence data of trees in the RAINBIO database as point vector data and a third layer containing country borders (42). All layers were reprojected to the same coordinate reference system, EPSG:4326—WGS 84. Next, the class of every data point in the RAINBIO database was determined. The information on classes was then added to the SmartWoodID database by counting all occurrences of a species and lower taxon in the RAINBIO database and counting the occurrence of each class. Finally, the classes were merged into larger classes, in order to give an easier overview on the occurrence of tree species and lower taxa in vegetation classes.

### Collecting the specimens

Creating a robust reference database requires the availability of many suitable wood specimens. Those wood specimens can be gathered by collecting specimens in targeted field expeditions, active timber harvest sites, lumber mills or other sites in the field. While such endeavours may faithfully capture the current data distribution, they can be logistically challenging and expensive to accomplish at large scale. A second source of information is institutional wood collections that have the advantage of having specimens readily available and that are, in some cases, the result of century-long collecting efforts. Wood collections that fit the requirements for building a robust reference image database, such as size, and reliability of specimens, are few in number though.

The Tervuren Wood Collection of the Royal Museum for Central Africa (RMCA, Belgium) was founded in 1898 to demonstrate the importance of African tropical timber for economic purposes. During the first half of the 20th century, the economic purpose has been gradually extended with a much broader scientific interest. Not only tropical species and lower taxa with commercial value but also any tropical African tree species and lower taxa that could be of interest in comparative wood anatomy or for the study of ethnographic objects were collected. From the middle of the 20th century and onwards, wood specimens from other continents were also incorporated in the collection (43–45).

Today, the wood collection has become the Belgian scientific reference collection for wood, containing ca. 81 000 specimens from 13 533 species and lower taxa with accompanying microtome sections, ca. 20 500 sets of thin sections in the three principal directions (15, 44, 45). Most of the species and lower taxa are represented by multiple samples, each from a different specimen.

The Tervuren Wood Collection holds 26 604 specimens of DRC tree species and lower taxa, which encompasses 30% of the total collection, thereby offering the most complete collection of reference material for wood identification of >2000 woody species and lower taxa from the DRC (timber trees, small trees, shrubs, dwarf shrubs and lianas). Those aspects of the wood collection create the unique opportunity to provide the robust reference database needed for building classification keys and CV-based wood identification tools by valorizing a vast collection of tree species and lower taxa with potential use as timber.

All DRC tree species and lower taxa, present in the Tervuren Wood Collection, are taken from the collection with at least four specimens per tree species and lower taxa. This ensures that variability in wood anatomical features between specimens of the same species and lower taxa is covered by the database. A typical wood collection sample is rarely intact because of the frequent presence of pin holes, traces of fungi attacks, cracks and other mechanical damage, making it difficult to produce clean polished surfaces that show the wood anatomical features without aforementioned anomalies. Specimens in the database that have such damage are not excluded from the database. They are included on purpose to ensure that the CV tools can learn to detect and ignore their presence. A lack of such damaged samples in the training data could cause the machine learning algorithm to explore such anomalies for recognizable and species and lower taxa defining characteristics.

### Sanding

The end-grain surfaces of the samples are sanded before scanning to ensure that all features, necessary for determination, are visible. The samples are stacked together with clamping screws to facilitate the process. The parameters of the machinery, more specifically angles and distances between the table, the sanding surface and the fulcrum, are set to be equal in order to ensure that every part of the surface is sanded at each grit.

The samples are first sanded using a belt sander at 100 grit to flatten the end-grain surface and subsequently using an eccentric sander. The end grain of the samples is pressed against the belt sanding surface with the appropriate amount of force at 1-s intervals, to prevent scorch marks, which can hinder the visibility of anatomical features.

Similarly, the end-grain surface of the samples is pressed against the eccentric sander while simultaneously performing lateral movements. Samples are sanded multiple times with gradually finer-grade sanding paper with each consecutive grit removing scratches from the previous grit and leaving shallower scratches. The eccentric sanding starts with a fine grade at 100 grit to remove all scratches of the belt sander and ends with an ultra-fine grade at 4000 grit at which point the end-grain surface is free of scratches and all macroscopically visible anatomical features are discernible with the naked eye or a ×10 magnifying glass.

At the end of sanding, a magnifying glass is used to check surface quality, and if necessary, both belt and eccentric sanding are repeated if any scratches are still present.

### Scanning

The sanded end-grain surfaces are scanned in order to visualize all macroscopically visible anatomical features. The scanning is performed using an Epson Perfection V750 Pro scanner using the SilverFast Ai Studio Version 9 software package. The scanner is calibrated twice a day with a 10 × 15 cm reflective Fuji Advanced Colour Calibration Target in order to ensure consistent results. A resolution of 2400 dpi or 95 pixel/mm was used in order to find a balance between storage need and a required resolution for observing all macroscopically visible anatomical features. A bit depth of 48 bit was selected to maximize the quantitative information (Red Green Blue values) on the natural colour of the wood. A typical image (TIFF file of 80 MB) will cover the wood anatomical end-grain structure of a surface of ∼7 cm long and 1–2 cm wide. The digital images cover more variability compared to sections of the usual size and provide opportunities for building elaborate classification keys and for performing substantial data augmentation (i.e. increasing image variability) for deep learning.

### Annotating

The resulting images are annotated by anatomical descriptions of the samples based on the list of macroscopic features (11) and the RGB values of the images. Twenty-nine of those standardized features are visible on a typical high-resolution scan of the end-grain surface (Table 2). Anomalies due to biological or mechanical impact that do not have a diagnostic value are also coded because they can hamper the identification process by non-experts or automated expert systems. It should be noted that the damage must not be too dominant on the specimen. During the identification process and the process of deriving the anatomical description, we found that specimens for which the damaged area was over two-third, a proper identification was often hard to obtain.

**Table 2.** The annotation content of SmartWoodID included in the database and derived from visual inspection of the sanded end-grain surface, encompassing 29 different macroscopic wood anatomical features (9) along with information on anomalies, mean colour values and wood density measurements

Structure	Property	Character	Character states	Macroscopic feature number IAWA
Growth rings	Growth rings	Growth rings distinct	Present/absent/variable	1
Vessels	Porosity	Diffuse porous	Present/absent/variable	3
		Semi-ring porous	Present/absent/variable	4
		Ring porous	Present/absent/variable	5
	Arrangement	Vessels in tangential bands	Present/absent/variable	8
		Vessels in radial pattern	Present/absent/variable	9
		Vessels in diagonal pattern (echelon)	Present/absent/variable	10
		Vessels in dendritic pattern (flame-like)	Present/absent/variable	11
	Grouping	Solitary and in radial multiples of 2–3 vessels	Present/absent/variable	12
		Exclusively solitary (≥90%)	Present/absent/variable	13
		Radial multiples of ≥4 common	Present/absent/variable/NA	14
		Clusters common	Present/absent/variable/NA	15
	Frequency	≤5 vessels per square mm	Present/absent/variable	16
		6–20 vessels/square mm	Present/absent/variable	17
		>20 vessels/square mm	Present/absent/variable	18
	Vessel diameter/ pore visibility	Small (not visible to the naked eye, <80 µm)	Present/absent/variable	19
		Medium (just visible to the naked eye, 80–130 µm)	Present/absent/variable	20
		Large (commonly visible to the naked eye, >130 µm)	Present/absent/variable	21
Axial parenchyma	Distribution	Diffuse-in-aggregates	Present/absent/variable	30
		Vasicentric	Present/absent/variable	31
		Lozenge-aliform	Present/absent/variable/unilateral	32
		Winged-aliform	Present/absent/variable/unilateral	33
		Confluent	Present/absent/variable/unilateral	34
		Banded	Majority wide/majority narrow/variable/absent	35
		Parenchyma in marginal or seemingly marginal bands	Present/absent/variable	38
		Reticulate	Present/absent/variable	39
		Scalariform	Present/absent/variable	40
Rays	Width	Ray visibility to the naked eye on the transverse surface	Rays not visible/all rays visible/only larger rays visible	43
	Rays per mm	Rays per mm	≤ 4 mm/5–12 mm/> 12 mm/NA	49
Anomalies	visible damage	insect holes	Present/absent/variable	–
		fungi	Present/absent/variable	–
		Mechanical damage	Present/absent/variable	–
Color	Red	Mean integer values	0–255	–
	Green	Mean integer values	0–255	–
	Blue	Mean integer values	0–255	–
Density measurement	Wood density	Density	[kg/m^3^]	–

The result is a list of 1700 tree species and lower taxa from the DRC, each with a description of the vegetation classes in which they grow, an indication of their commercial value and their threatened status in 2022 according to the IUCN Red List (46), the CITES (47) and the European Union and Trade in Wild Fauna and Flora (48). Of these 1700 species and lower taxa, 1190 species and lower taxa are present in the Tervuren Wood Collection and are used to create images and annotations on the macroscopic anatomical features with at least four specimens available for all species and lower taxa, thereby resulting in 4740 surfaces to scan. The pursued number of four specimens per species and lower taxon was chosen to correspond to the available number of collection specimens in the Tervuren Wood Collection. In addition, it is common practice in wood anatomical assessments to base species and lower taxa descriptions on a relatively small amount of specimens.

### Quality control

A database with reliable reference material is the backbone of any application to identify a specimen in a taxonomy system. A first important aspect of reliability to address is the need for specimens to be correctly identified. If misidentified, it would cause the interpreter, being either a wood anatomist or a machine learning model, to focus on different distinguishing characteristics for said species and lower taxa, potentially resulting in misidentification. The Tervuren Wood Collection contains specimens that were collected during field missions. During many field missions, herbarium material was also collected and stored in the collection of the Meise Botanic Garden (49). Specimens with reliable herbarium vouchers in the Meise Botanic Garden are primarily selected to ensure the reliability of the specimens. When specimens with herbarium material are not available, specimens from reliable collectors are chosen. Next, specimens are compared with descriptions in the InsideWood database in order to maximally avoid misidentification during annotation. Because the features checked during annotations are all macroscopic for hardwood identification listed by the IAWA and visible on the end-grain surface, the InsideWood database provides the perfect reference tool for checking the occurrence of IAWA features and the correct identification of wood collection samples. An unknown wood specimen can be a species and lower taxon not present in the database (34). The Tervuren Wood Collection contains multiple samples of most species and lower taxa, and for the image database that we present, four specimens of each species and lower taxon are selected. In order to ensure a good number of high-quality specimens, specimens with a large end-grain surface are preferred as they contain more information. Wide branches and stem disks are also included if possible, as they have a large end-grain surface along with extra information, like pith and differences between heartwood and sapwood. Twigs and branches are only included if no other specimens are available because the smaller area of the end-grain surface consists of juvenile wood mainly and does not show the diagnostic features used in routine wood identification on the variability of anatomical features. Some of the different macroscopic features can also differ between different parts of the tree, for example, the size of vessels will be substantially larger in the stem compared to branches and especially twigs (50).

### Technical description of the database and functionalities

The specimen-based database with the collected observations is made accessible online by incorporating it in an IIIF environment, where IIIF stands for International Image Interoperability Framework for presenting and annotating content such as images and audio-visual files (51, 52). This framework was selected due to the potential it has for sharing data in a way that allows viewing, comparing, manipulating and annotating images in an environment that is easily accessible. The SmartWoodID database within the IIIF contains new high-resolution scans of wood and accompanying metadata such as geographical origin, accepted taxonomy according to the WCVP, descriptions of their anatomical features, the mean RGB values of intact wood and the density measurements.

The IIIF environment is implemented with the Image Application Programming Interface (API) and the Presentation API only, with plans to add the Content Search API in a later stage. The Image API defines how image servers deliver pixels to a viewer, and the Presentation API adds metadata and structures to these images, defining how they appear in IIIF-compliant viewers. This is done through an IIIF Manifest, a JSON file. These JSON files are generated with a custom Python script that fetches all relevant information from the database.

**Figure 3.**
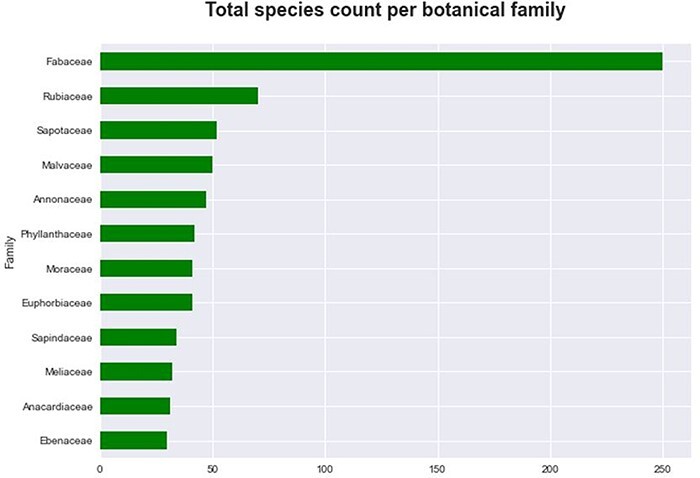
The 12 largest botanical families of tree species ranked according to the highest number of species in the database.

The Manifest file is presented by an IIIF-compliant viewer online. There are several (open-source) viewers available, each with its own use case. Since IIIF is all about interoperability, the Manifest file can be reused potentially within different viewers. Mirador (53) was selected as the primary viewer as it is an open-source, highly configurable and extensible multi-window image viewing platform that allows researchers to view, zoom, rotate and compare image-based resources, making it both educative and useful for experts and wood enthusiast alike. The viewer is also not limited to viewable specimens but can view and compare any IIIF-enabled resource available, facilitating research across collections and institutions.

In a later stage, annotations will be added to display the macroscopic wood anatomical characteristics in order to visualize them for educative purposes and to potentially include them in identification applications such as classification keys and AI using object identification to recognize and quantify wood anatomical properties.

## Results

### Taxonomic coverage

The database contains 1190 tree species and lower taxa present in the Tervuren Wood Collection encompassing 421 genera and 94 families. The family with the largest number of species and lower taxa in the database is *Fabaceae*, covering 250 species and lower taxa and 21% of all DRC tree species and lower taxa. The fact that *Fabaceae* is the most diverse tree family is not surprising given that *Fabaceae* or *Leguminosae* is the third most diverse plant family after the (primarily herbaceous) families *Asteraceae* and *Orchidaceae* (54). The second and third most occurring families are the *Rubiaceae* and the *Sapotaceae*, covering significantly less species and lower taxa with 70 and 52 species and lower taxa and 6% and 4% of all DRC tree species and lower taxa, respectively. The 12 most occurring families are shown in Figure 3 and encompass 60% of the 1190 species and lower taxa with not <30 species and lower taxa per family. *Ficus* is the genus with the most species and lower taxa at 2.69% of all species and lower taxa closely followed by *Diospyros* at 2.35% of all species and lower taxa.

### Geographical coverage

**Figure 4.**
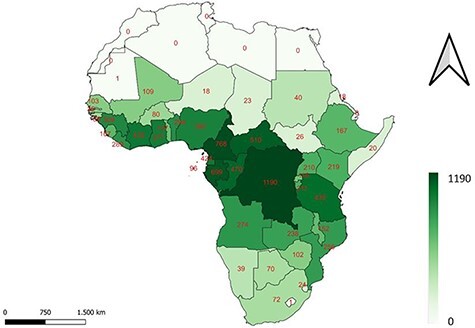
A map of the African continent, indicating the number of DRC tree species present in each country. The darker the colour, the more species present.

Figure 4 shows in which countries on the African continent the tree species and lower taxa from the SmartWoodID database are growing. The colour intensities represent the number of tropical tree species and lower taxa of the DRC present in that country according to the RAINBIO database, ranging from 0 to 1190. There is a gradient moving away from the equator, as less DRC tree species and lower taxa occur further north or south of the continent, which is logical given the tropical boundaries. Given that the DRC is a large country covering a wide spectrum of phytogeographical regions, this obviously results in a large number of vegetation classes (Table 3) also present in neighbouring countries harbouring many of the same species and lower taxa. The DRC also covers the majority of the Guineo-Congolian regional centre of endemism, one of the largest and most biodiverse regions of Central Africa, that encompasses both ‘Moist Central Africa’ and ‘Wet Central Africa’ (55, 56). Those facts further support that the DRC is a relevant geographically delineated area to produce a robust reference database of images and wood anatomical descriptions for species identification.

### Threatened status

Only 23 of the 1700 DRC tree species and lower taxa are included in the Checklist of CITES (47), which is shown in Table 4. All those tree species and lower taxa are listed in appendix II of the Checklist of CITES and annex B of the European Union and Trade in Wild Fauna and Flora regulation (48) and are therefore considered species and lower taxa not necessarily threatened with extinction now, but they may become so unless trade is closely controlled. For each species and lower taxon, the IUCN Red List Category is presented with sometimes remarkable results as some species and lower taxa are of least concern according to the Red List, while the Checklist of CITES includes them. One reason for this might be the year of the last assessment by the IUCN. *Pterocarpus tinctorius* is a good example of this, as it was last assessed in 2017. It was appended to appendix II of the CITES at the Nineteenth meeting of the Conference of the Parties (CoP19) in Panama in 2022 because more recent assessment showed the heightened risk of extinction due to trade (57). Outdated assessments by the IUCN are not the only reason that a species and lower taxon might be appended to CITES. *Afzelia bella*, for example, was last assessed in 2019, which showed that the population remains stable on a global scope and that the species is of least concern. It was however added at the CoP19 due to being a look-a-like species for threatened species such as *Afzelia africana, Afzelia bipindensis, Afzelia pachyloba* and *Afzelia quanzensis* (58).

**Table 3.** An overview of all general vegetation classes in the DRC defined by Fritz *et al.* (2003) (41) with the total number of tree species (available in the SmartWoodID database) in each vegetation class and the percentage of threatened species present in each vegetation class

General vegetation class	Number of tree species per class	Percentage of threatened species	Percentage of species at lower risk	Percentage of species with deficient data on threatened status
Closed forest	886	8	16.4	64.4
Altitudinal forest	302	7	17.5	68.2
Edaphic forest	323	2.8	11.5	71.5
Woodland	543	4.2	10.7	68.5
Shrub land	355	4.5	9.6	71.8
Savannah	304	3.9	9.2	73.4
Urban areas	178	3.9	6.2	73
Desert	0	0	0	0
Water bodies	306	3.3	13.7	69.9

**Figure 5.**
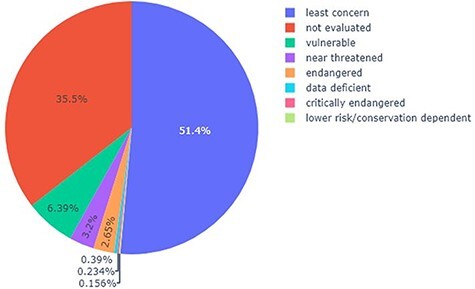
Pie chart showing the percentage of each category (according to the IUCN Red List) present in the list of trees in the DRC, capable of providing timber.

Figure 5 shows how many of the listed species and lower taxa belong to each of the nine IUCN Red List Categories. Only 8 of the 10 categories are present in the list of DRC timber species and lower taxa as it contains no species and lower taxa that are extinct or extinct in the wild. Nine per cent of all listed species and lower taxa belong to one of the three threatened categories (vulnerable, endangered and critically endangered). The list contains 85 vulnerable species and lower taxa such as *A. bipindensis, Baillonella toxisperma* and *Entandrophragma utile*. Thirty-six species and lower taxa are classified as endangered such as *Millettia laurentii, Pericopsis elata* and *Autranella congolensis*. Only three species are considered critically endangered, *Beilschmiedia donisii, Elaeophorbia drupifera* and *Warneckea superba*. Sixty per cent of all species and lower taxa are of lower risk, more specifically near threatened, least concern and conservation dependent. About 43 species and lower taxa are near threatened like *Milicia excelsa, Entandrophragma angolense* and *Dialium pentandrum*. Half of the listed species and lower taxa are of least concern to being threatened. Two species are conservation dependent and six species belong to the category data deficient because there are little data about their distribution and/or abundance. The remaining 35% of the listed species and lower taxa have not been evaluated yet.

**Table 4.** All tree species in the DRC that are appended to appendix II of Checklist of CITES Species (47), along with their respective IUCN Red List Category, IUCN population trend and last year they were assessed (46)

Species name	IUCN Red List Category	Population trend	Last year assessed by IUCN
*Afzelia africana*	Vulnerable	Decreasing	2019
*Afzelia bella*	Least concern	Stable	2019
*Afzelia bipindensis*	Vulnerable	Unknown	1998
*Afzelia pachyloba*	Vulnerable	Unknown	1998
*Afzelia peturei*	Vulnerable	Decreasing	2019
*Afzelia quanzensis*	Least concern	Decreasing	2019
*Alsophila camerooniana*	Least concern	Unknown	2016
*Dalbergia nitidula*	Least concern	Stable	2018
*Euphorbia abyssinica*	Not evaluated	Unknown	
*Euphorbia ingens*	Least concern	Stable	2018
*Euphorbia teke*	Not evaluated	Unknown	
*Guibourtia demeusei*	Near threatened	Decreasing	2020
*Khaya anthotheca*	Vulnerable	Unknown	1998
*Khaya grandifoliola*	Vulnerable	Unknown	1998
*Pericopsis elata*	Endangered	Decreasing	2020
*Prunus africana*	Vulnerable	Decreasing	2020
*Pterocarpus angolensis*	Least concern	Decreasing	2018
*Pterocarpus lucens*	Least concern	Stable	2010
*Pterocarpus rotundifolius*	Least concern	Stable	2018
*Pterocarpus soyauxii*	Not evaluated	Unknown	
*Pterocarpus tessmannii*	Near threatened	Unknown	2020
*Pterocarpus tinctorius*	Least concern	Decreasing	2017

### Economic value

The list of DIAF 2017, summarizing all tree species and lower taxa present in DRC forests, also gives an indication of their current economic value (38). The species and lower taxa are divided into four categories: I (commercially exploited species and lower taxa) consisting of 26 species and lower taxa, II (species and lower taxa with potential to be used commercially) consisting of 19 species and lower taxa, III (species and lower taxa with potential to be used commercially, but with few knowledge on their material properties) consisting of 42 species and lower taxa and IV (species and lower taxa with no known economic value) which is the majority of the database at 1613 species and lower taxa. Categories I, II and III are considered as economically important classes due to the use or potential use of these species and lower taxa. A study that analysed 31 logging concessions in the five International Tropical Timber organization member countries of the Congo Basin was able to determine the 35 timbers from tropical Africa which amount to 94.2% of the total timber volume produced annually in the Congo Basin (59). Of those 35, only three species do not occur in the DRC according to the database. Those three species, *Distemonanthus benthamianus, Brachystegia cynometroides* and *Testulea gabonensis*, account for <3% of the total timber volume produced in tropical Africa. This shows that the DRC is rich in commercial species and lower taxa, although it is important to note that those species and lower taxa do not necessarily show the same abundance in the DRC compared to other countries. An example of this is *Aucoumea klaineana*, the most traded species in the Congo Basin, which is only sparsely present in the DRC because it mostly grows in West-Central-African countries such as Gabon (59).

## Discussion

### The DRC and its representativeness

The choice of the DRC as a basis for developing the first version of the SmartWoodID database is affirmed by the geographical analysis as the DRC is a large country with a large variety of vegetation types. This ensures that a large part of the species and lower taxa from the DRC are also present in neighbouring countries, making the database relevant in an international context. Illegal logging and fraudulent deliveries of timbers are not geographically limited to DRC. These malpractices with the same species and lower taxa happen in other African countries, and therefore, wood identification tools for all DRC timber-producing species can help combat illegal logging across borders.

Total species richness is however not the only parameter to select the DRC as the geographic area of choice for the database. The choice also depends on the type of species, their economic value and threatened status. Some species are protected by the CITES convention (47) and The European Union and Trade in Wild Fauna and Flora (48) while also being highly interesting for commercial use such as *A. bipindensis, Khaya anthotheca, Pterocarpus soyauxii* and *P. elata*. If such species are logged illegally, it can lead to severe population loss and even impact species that are not currently threatened or that are of least concern due to the damage to precious forest stands in search for valuable trees. A reference database for wood recognition should therefore contain the most prominent exploited timbers, as a wood identification tool will frequently encounter commercial and threatened timbers. The SmartWoodID database contains 32 of those 35 commercial timbers, showing that the DRC is host to almost all highly commercialized timbers from the Congo Basin (59). The large amount of commercial species and threatened species makes the SmartWoodID database and wood identification tools derived from it also usable in importing countries. This last aspect is particularly important as this is where regulations go in effect and where wood identification techniques must be applicable on a systematic basis.

### Opportunities of the SmartWoodID database

An image database with information on wood anatomical features has clear goals to aid in identifying the botanical taxon of species, by serving as reference material for distinguishing them. In this regard, an image reference database with information on wood anatomy must be complete regarding its content. It should therefore not only aim at encompassing all species that logically can be encountered in trade but must also maximally cover all possible types of irregularities.

Containing all species is particularly important because the value of identification tools depends on the completeness of its reference data. Especially for a species-rich country like the DRC, databases should be as large as possible to reduce the risk that a tool is only developed for a small part of the flora and that a positive identification is not possible only because many species are not included in the database. Moreover, it is very unlikely that foreign species are being imported in the DRC, so the database should purely focus on the maximum of species present in the DRC.

Wood samples most often contain many irregularities visible on a wood surface due to its nature as a natural product that is subservient to the growing conditions of a tree or post-growth incidences such as mechanical damage or damage by insects or fungi. Those irregularities hamper a smooth identification process and are a main reason why expert knowledge is needed to distinguish between diagnostic characteristics and other features. This makes this information particularly relevant for anyone performing wood anatomical assessments in the field. Any tool used to identify wood with anatomy, such as classification keys or AI, must therefore take the irregularities into account. It is the inclusion of data on such irregularities that distinguish the image collection of the Tervuren Xylarium compared to other large image databases that contain high-resolution images of the end-grain surface, such as the database of the XyloTron system (60).

Another difference between SmartWoodID and other databases is the large end-grain area scanned. A large end-grain surface contains a maximum of information on variably occurring macroscopic features. The amount of anatomical information is therefore higher and available to be used in research and development of identification tools. The variability of wood anatomical characteristics between specimens must also be considered as growth conditions and genetic traits can lead to varying wood anatomical features. Therefore, databases should contain information on several specimens for each species in order to cope with the natural variability of wood between individual trees.

The information on variably occurring macroscopic features and the recorded data on irregularities enrich the SmartWoodID database and ensure its robustness needed to create tools capable of aiding fieldworkers in accurate identification.

## Conclusion

The SmartWoodID image database will offer new opportunities for developing identification systems based on recognition of diagnostic wood anatomical features. This database is unique since it covers a large number of African tree species and lower taxa of which the macroscopic structure is visualized and described. The Tervuren Wood Collection provides this thanks to its heritage of collecting reliable reference material over the span of more than a century. A total of 71% of all DRC tree species and lower taxa, listed in DIAF (2017) (38), are currently available within the Tervuren Wood Collection. The first version of the SmartWoodID image database that is presented here consists of a set of 1190 timber species and lower taxa present in the DRC forests (38, 39). The database focuses on the macroscopic anatomical features that can be encountered on a high-resolution scan of end-grain wood surface. The database accounts for irregularities and natural variability, using multiple specimens with large end-grain surfaces. This makes it a robust reference database for research on wood in general and will allow the development of tools for aiding in law enforcement to combat illegal logging.

## Supplementary Material

baad034_Supp

## Data Availability

De database and all its data will remain publicly available for a minimum of two years starting from the day of publication.
