# Supplementary material for: SmartWoodID—an image collection of large end-grain surfaces to support wood identification systems
Source: Database (Oxford). 2023 May 13;2023:baad034. doi: 10.1093/database/baad034 (PMC10182821; doi:10.1093/database/baad034)
Supplement: baad034_Supp [file baad034_supp.zip › suppl_data/Bulletpoints.docx]

- Wood identification is a key step in the enforcement of laws and regulations aimed at combatting illegal timber trade.
- Robust wood identification tools, capable of distinguishing a large number of timbers, depend on a solid database of reference material.
- Specimens from the Tervuren wood collection, one of the large institutional wood collections around the world, are used as source of tree species data with potential application as timber.
- Here we present SmartWoodID, a database of high-resolution optical scans of the end-grain surfaces enriched with expert wood anatomical descriptions of macroscopic features.
- SmartWoodID can serve as annotated training data to develop interactive identification keys and artificial intelligence for computer vision-based wood identification.
